# Mountain Child: Systematic Literature Review

**DOI:** 10.1007/s10995-016-2051-8

**Published:** 2016-07-11

**Authors:** Annie Audsley, Rebecca M. M. Wallace, Martin F. Price

**Affiliations:** 1 Edinburgh, UK; 2 The Robert Gordon University, Aberdeen, UK; 3 University of the Highlands and Islands, Perth, UK

**Keywords:** Literature review, Mountain, Child, Wellbeing, Poverty, Health, Nutrition, Education, Child mortality, Child labour/child labor

## Abstract

*Objectives* This systematic review identifies and reviews both peer-reviewed and ‘grey’ literature, across a range of disciplines and from diverse sources, relating to the condition of children living in mountain communities in low- and middle-income countries. *Findings* The literature on poverty in these communities does not generally focus on the particular vulnerabilities of children or the impact of intersecting vulnerabilities on the most marginalised members of communities. However, this literature does contribute analyses of the broader context and variety of factors impacting on human development in mountainous areas. The literature on other areas of children’s lives—health, nutrition, child mortality, education, and child labour—focuses more specifically on children’s particular vulnerabilities or experiences. However, it sometimes lacks the broader analysis of the many interrelated characteristics of a mountainous environment which impact on children’s situations. *Themes* Nevertheless, certain themes recur across many disciplines and types of literature, and point to some general conclusions: mountain poverty is influenced by the very local specificities of the physical environment; mountain communities are often politically and economically marginalised, particularly for the most vulnerable within these communities, including children; and mountain communities themselves are an important locus for challenging and interrupting cycles of increasing inequality and disadvantage. While this broad-scale review represents a modest first step, its findings provide the basis for further investigation.

## Significance

The review compares literature from across disciplines and geographical locations to look at the effect on children of growing up in a mountainous environment. The significance is in the finding of common themes across the literature, which tend to confirm the broad applicability of Ives’ description of a “mountain problematique” [27, p. 10].

*What this study adds*: Geographic specificity is important in understanding mountain poverty and most of the literature reviewed here focuses on specific geographic areas and the local determinants of poverty. However this review takes a comparative perspective in order to examine whether mountainous environments also have a general effect on child poverty.

## Context, Aim and Objectives

Mountains cover 22 percent of the Earth’s land surface and are home to 13 percent of its population [54]. Of these 915 million people (in 2012), 39 percent in developing countries are considered vulnerable to food insecurity: a 30 percent increase since 2000 [26], while the mountain population has increased by only 16 percent. Arguably, therefore, the vulnerability of mountain people is increasing, and the “mountain problematique” identified by Ives [27, p. 10, 28] two decades ago is intensifying. Ives argued that this situation is due to the combination of marginal ecosystems; remoteness and cultural or ethnic distinctiveness, sometimes compounded by environmental degradation; natural hazards; globalisation; and, often, conflict. The disproportionate effect of poverty on women and, in turn, on children leads to the mountain girl child being the “most vulnerable member of humankind” [27, p. 29]. Ives [27] urgently called for further research on the situation of children in mountain communities.

The aim of this review was to test whether the existing literature confirms Ives’ view that children in mountainous environments in low- and middle-income countries are disadvantaged compared with lowland children in the same country or region. Based on his description of the “mountain problematique”, a wide variety of interconnected factors impacting on mountain children was anticipated. The objective was to identify and gather evidence which could provide insights into how growing up in a mountainous environment affects children. Specifically, the review hoped to identify, first, how and why such children may be materially affected and, second, crosscutting themes in authors’ discussions of these impacts. The project’s scope was broad rather than deep: the intention was to use literature from different disciplines to identify the range of factors which could impact on children’s wellbeing, and to describe themes which recur across disciplines and world regions, in order to provide a context within which to understand the condition of children in mountainous environments and suggest themes for further investigation.

## Methods

Web searches were used to identify documents from a variety of sources: peer-reviewed journals covering a range of disciplines, and reports from research centres, Non-Governmental Organisations (NGOs) and United Nations (UN) agencies. Search terms were usually “child AND mountain OR highland”, the only exceptions being databases which focus on either children or mountains. For example, the search term used for the Mountain Forum online library was simply “child.” An iterative approach [43] was used, “snowballing” searches to look at references from literature that had already been identified and sourcing other papers by known authors. The full search plan for the review is included as an “Appendix”.

Eligibility criteria for inclusion in the review were broad. Any article or report, published or unpublished, which revealed anything about a relationship between mountainous environments and the condition of children was included. Documents that simply mentioned that the study took place in a mountainous environment or did not engage with the question of how the mountainous context impacted on children’s wellbeing were excluded from the review. The selection was limited to studies in English written since 2000, both to provide a contemporary view and because of limited resources. Documents which met the eligibility criteria were selected for review by reading the abstract and, where necessary, scanning the entire document.

The initial web searches identified 165 documents, from which 68 were selected for the review by reference to the eligibility criteria. The table below gives a breakdown of documents which were selected, according to topic and type of document (Table 1).

**Table 1:** Document sources and topics

Topic	Peer reviewed journal articles	UN/Gov/intergov org publications	NGO publications	Development bank publications	Other (book chapters, partnerships)	Total
General	0	1	2	1	0	4
Poverty	0	4	1	3	4	12
Health	6	1	3	0	1	11
Nutrition	13	2	0	0	1	16
Child mortality	6	0	0	0	0	6
Education	4	2	4	2	2	14
Child labour	3	2	0	0	0	5
Total	32	12	10	6	8	68

Given the diverse sources, analysis or comparison of empirical data was not relevant; instead, the discussion is based on the authors’ conclusions and observations. For each document, notes were made and brief summaries written, drawing out any evidence or discussion which addressed the impact of mountainous environments on children. A spreadsheet was then used to categorise documents into broad topics (see table above) and themes (driving forces). These themes were assessed according to the frequency with which they recurred in the literature, particularly across documents on different topics. For example, marginalisation featured in discussions on poverty, health, nutrition, education and child labour.

The review was carried out by two individuals. One completed the preliminary search, selected documents for review, read and took notes. The two individuals then worked together to identify themes, discuss conclusions, and write this paper.

## Discussion

This discussion begins with an examination of literature on different topics: poverty, health, nutrition, child mortality, education, and child labour; then going on to identify some crosscutting themes. References are cited with regard to specific themes. They are also included in the list of references at the end of the article.

### Poverty

Literature on mountain poverty gives contextual and explanatory frameworks for understanding the situation of children in mountain communities. No literature focusing specifically on both mountainous environments and child poverty was identified, other than [27, 28]. However, the use of child indicators, such as school enrolment and infant mortality rate (IMR), enables some analysis of the effects of mountainous environments on child poverty.

Findings in the literature are varied, and suggest that the effects of mountainous environments on child poverty are not straightforward; many other factors have an influence. Most documents do conclude that mountainous environments have a negative impact on the economic situation and human development of mountain communities; some literature explicitly theorises the concept of *place*, interpreting geographic location as the primary determinant of poverty and well-being. Explanatory factors include remoteness and geographic conditions (Bird et al. [8], introducing a typology of “remote rural areas”; Ssewaya [62], on a mountainous area of Uganda; Jolliffe [29], on Afghanistan; UNICEF [64], on Lesotho; Gerlitz et al. [22], looking at determinants of mountain poverty across the Hindu Kush Himalayas); cultural or ethnic distinctiveness or social-political exclusion (Baulch et al. [6], Dang 15], both on Vietnam); environmental degradation [22]; and lack of access to external markets (Joshi [30] on Himalayan states of India). All of these factors can be exacerbated by the effects of rapid economic, social and climatic change (Walker [67], on childhood vulnerability to climate change in mountainous areas of Vietnam; Gerlitz et al. [22]). The fragile balance can also be more catastrophically upset by natural disasters or violent conflict, both of which are often characteristic of mountainous regions [27], with impacts on all aspects of children’s lives (Asian Development Bank and World Bank [2], on the impact of the 2005 earthquake in Pakistan; Mohamed et al. [42], on the impacts of violent conflict in the central highlands of Papua province, Indonesia).

Other studies present a more varied picture, illustrating the complexity of mountain poverty: mountainous areas are not always poorer than lowlands. For example, a report on poverty in Nepal [3] finds that the Mountain and Terai (lowland) regions consistently have the worst child poverty indicators and the Hill (mountain) regions, by most measures, fare better. Similarly, a study of poverty in four sites in Ethiopia for Oxfam [65], covering both lowlands and highlands, described the former as “remote”, with access to health services and schools no better than in rural highland areas.

The literature cited above shows how mountainous environments can often have negative impacts on human development across a wide range of indicators, but none of these documents thoroughly explore the particular vulnerability of children in these environments. The literature reviewed in the following sections does focus more specifically on children, but often without the wider contextualisation provided by the literature on poverty.

### Health

The literature on health services reveals a number of problems regarding access to these services in mountain communities, with impacts on children’s health. Perhaps most common is the impact of geographical remoteness on access to such services (Singh [61], on the Tibetan Autonomous Region; Gyaltsen et al. [23], on Qinghai Province, China; literature review on access to and use of health services in mountainous regions by Byrne et al. [9]. In contrast, one study from Ethiopia describes greater remoteness and worse health outcomes in the lowlands compared to the highlands [55]. Remoteness is not the only factor leading to poor access to services: political priorities and individuals’ decisions over the use of services are also relevant (Singh [61] and Morris [44], on uptake of health services in Honduras; Van Vo et al. [66], on the use of maternal health care services in Vietnam; Rheinländer et al. [52], on care-seeking practices in Vietnam; Oxfam [47], on provision of healthcare services in Nepal; Byrne et al. [9]).

Children living in mountain communities also face risks to their health related to their location, such as increased risk of pneumonia and respiratory infections at high altitudes (Khan et al. [31], on Pakistan; literature review on child health at altitude by Niermeyer et al. [45]); risks of injury (Shi et al. [58], on South-west China); or the increased risks of natural disasters (Chohan [12], on the impact of flooding in Khyber Pakhtunkhwa and Punjab, Pakistan). Other risks to children’s health relate to the wider context of poverty in mountain communities, as discussed above [23].

### Nutrition

Many studies identify a nexus between mountainous areas and child malnutrition (Leonard et al. [35], Larrea and Kawachi [33]; Rogers et al. [53], on Ecuador; Harris et al. [25], Dang et al. [16, 17], on Tibetan children; San Miguel et al. [56], on Bolivia; Dutta and Pant [19], on the Garhwal Himalayas; Larrea et al. [34], on the Andes; Miyoshi et al. [41], on the Lao PDR; Wang et al. [68], on rural North West China). While unfavourable geographical conditions seem to be a large contributory factor [56, 19, 34, 41, 68], other factors, such as lack of healthcare facilities [25], economic inequality [33], maternal education [68] and cultural factors [17] also have effects. The impacts of poverty and hardship on early feeding practices [32] are also relevant.

Mountainous regions tend to be characterised by deficiencies of micronutrients, primarily iodine and iron, and specific deficiencies are associated with particular mountainous areas [26]. Micronutrient deficiencies are associated with significant health problems (Zimmermann et al. [71], on the interaction between iodine and iron deficiency in a mountainous region of Côte d’Ivoire; Oberlin et al. [46], on goitre and iodine deficiency in Afghanistan; Singh and Choudhary [60], on the health impacts of anaemia on teenage girls in the Himalayas; Counter et al. [13], on the impact of anaemia and lead exposure on the hearing of children living at high altitude in the Andes).

### Child Mortality

Child mortality (particularly infant mortality) is often used as an indicator of poverty or health. Of the literature discussed above, several studies suggest that high IMRs are associated with mountainous areas. For example, studies in the Tibetan Autonomous Region [61] and Vietnam [15] both describe IMRs that are significantly higher in mountain areas than at national levels in the respective countries.

High rates of child mortality often appear to be related to generally poor levels of human development which, in turn, characterise mountainous areas (Manandhar et al. [39], on Nepal; Wellhoner et al. [69], on Qinghai Province, China). A study conducted in Ladakh [70], found that altitude *per se* is a specific factor, combined with the fact that the population has a relatively short ancestry in the area and may not have developed genetic adaptations to living at altitude. In contrast, Chin et al. [10] find that in Nepal, altitude is statistically a protective factor, when controlling for other known factors.

De Hilari et al. [18] examine infanticide in two highland villages in Bolivia and suggest that it could be related to poverty and isolation.

### Education

Children can face many problems in receiving education in mountainous areas. Possibly most notable are the physical accessibility and quality of schools in remote areas (Fung [21], on mountainous areas of China; Postiglione [50], on Tibet; Siaens and Goyal [59], on Bhutan), which can be exacerbated by other overlapping vulnerabilities (Barriga [5], on access to education for children with disabilities in Nepal; Choden [11], finds that poverty affects school attendance and enrolment in Bhutan). However, a meaningful education also requires that schools are linguistically and culturally appropriate (Middleborg [40], on Ratanakiri Province in Cambodia, Postiglione [50], on Tibet; Bangsbo [4], on the Tibetan regions of Sichuan and Qinghai Provinces). Political will and policy frameworks allowing for diversity are vital to ensure the provision of education which is accessible, appropriate and of good quality [40]. Where government acceptance of diversity is lacking, education can become an arena of conflict over identity (Arquiza [1], on the education of ethnic minority children in a mountainous region of the Philippines).

A number of studies examine the impact of gender equality on school enrolment or attendance. Their findings are quite mixed: while some studies associate gender inequality with mountainous areas (UNDP [63], on the Lao PDR; Oxfam [48], on Afghanistan; Parker et al. [49], on Nepal), others find that national-level characteristics are more influential than the difference between mountain and lowland areas (Hannum and Adams [24], on Gansu Province in China where gender inequality is declining; Lloyd et al. [37], Lloyd and Ghuman [36], on Pakistan where the gender gap is wide in poor, rural, and remote areas of both mountains and lowlands).

### Child Labour

Child labour is another major factor impacting on children’s educational outcomes, health and wellbeing. Several studies describe the prevalence of child labour in mountainous regions and its association with poverty, remoteness and marginalised communities, in particular those communities experiencing increased pressures from rapid economic or environmental change (Sherchan [57], on children in mountain communities in Nepal; Dammert [14], on child labour in Peru following a rapid reduction in coca production). Prasun and Kainthola [51] describe how, in circumstances of extreme inequality and marginalisation in a remote area of Uttarakhand, India, children are vulnerable to bonded labour and trafficking. In contrast, another study in Uttarakhand [38] suggests that environmental degradation which threatens traditional livelihoods has led to an increased perception of the need for education and in turn, increased school enrolment.

## Themes

The discussion now turns to certain characteristics of mountainous environments which were recurring themes across the reviewed documents.

### Physical Environment

The literature makes clear that mountain communities are affected by very specific local conditions and that spatial or geographic factors are key determinants in mountain poverty. Several recent studies have been able to map poverty indicators at a much more local level than previously, enabling a better understanding of specific geographic factors [53, 10, 68]). Mountain poverty is therefore complex and varied, and an understanding of the specific local conditions is vital in order to help develop appropriate interventions (Bayoumi [7], on a community-based birth registration project in the Nuba mountains in Sudan; Walker [67]; Gerlitz et al. [22]).

### Marginalisation and External Influence

Many studies state that mountain communities tend to be politically and economically marginalised, often because they are ethnically or linguistically distinct from lowland urban and political centres [4, 20, 25, 33, 52]. Such marginalised communities are more vulnerable to the additional stressors created by economic, social or environmental and climactic changes (e.g. [30], on the negative influence of market liberalisation; [19], on the downward spiral of poverty and environmental degradation). Many studies also consider intersecting vulnerabilities based on caste, class, gender and—of particular relevance here—generation, creating conditions of particular disadvantage for the most vulnerable members within mountain communities [20, 22, 49, 67]. Consequently, many advocate a child rights based approach [47, 48, 61, 67] and investment in improved accessibility, quality, and cultural appropriateness of services [1, 9, 50, 52, 66, 69].

### Mountain Communities

Mountain communities themselves can contribute not only to the perpetuation of disadvantage but also to its interruption. Isolated communities can be associated with harmful cultural practices (eg infanticide: De Hilari et al. [18]) or traditional beliefs and social relations which maintain inequality and disadvantage among sections of the community. For example, the negative impact on children’s wellbeing of gender inequality is a frequently recurring theme [20, 47–49, 52, 60, 63, 66]. However, much of the literature points to the agency of communities and of children themselves in challenging traditional practices and beliefs [49, 22] and in adapting to external pressures such as climate change [67] or constructing meaning and self-respect within their current situation [20]. Going beyond the scope of this review on the impacts of mountainous environments on children’s material wellbeing, the recurring theme of the strength and importance of mountain communities and, in particular, Dyson’s work, suggests that an alternative line of investigation could look at non-material effects such as mental health and wellbeing, constructed meanings, and identities.

## Conclusions

This review has examined literature across many disciplines and publications. Peer-reviewed articles range from statistical analysis of health outcomes to ethnographic and qualitative studies looking in greater depth at children’s individual experiences. The reports of NGOs and UN agencies often have a more straightforward analysis based on their particular agendas. While bias is a possibility in some of these publications, nonetheless they provide insights into the condition of children in mountainous environments.

The literature tends to support the view of Ives [27, 28] that children are, in general, negatively affected by living in mountainous environments in low- and middle-income countries. The literature on poverty does not generally focus on the particular vulnerabilities of children or the impact of intersecting vulnerabilities on the most marginalised members of communities. Exceptions were Walker [67], Gerlitz et al. 22], who account for intersecting vulnerabilities in their analyses. However, the literature on poverty does contribute analyses of the broader context and variety of factors impacting on human development in mountainous areas.

The literature on other areas of children’s lives—health, nutrition, child mortality, education, and child labour—is more specifically focussed on children’s particular vulnerabilities or experiences. However, it sometimes lacks the broader analysis of the many interrelated characteristics of a mountainous environment which impact on children’s situations. Nevertheless, themes were identified which recur across many disciplines and types of literature and point to some general conclusions: mountain poverty is influenced by the very local specificities of the physical environment; mountain communities are often politically and economically marginalised, which is exacerbated for the most vulnerable within the communities, including children; and mountain communities themselves are an important locus for challenging and interrupting cycles of increasing inequality and disadvantage.

The limitations of this study are that it sacrificed depth of analysis, comparability of results, and assessment of the quality of individual studies for a broader view of the context and the identification of themes which cut across disciplines. The global perspective adopted is also at the cost of investigating the geographical specificity of any one region. The rationale for this wide scope was to focus on the impact of mountainous environments *per se* rather than to look in depth at any one community or region, as a wide range of examples was needed to test the broad applicability of Ives’ postulate. Nevertheless, this review represents a modest first step, providing a range of findings which provide the basis for further investigation.

## Appendix: Online Searches

**Table:**

Organisation	Web address	Search terms
*Journals/Databases/Research Centres*		
Taylor Francis Journals	http://www.tandfonline.com	Child AND mountain OR highland
Oxford Journals	http://services.oxfordjournals.org	Child AND mountain OR highland
Bio One Online Journals	http://www.bioone.org/action/doSearch	Child AND mountain OR highland
Mountain Forum	http://www.mtnforum.org/publications	Child
Lancet	http://www.thelancet.com	Child AND mountain OR highland
Elsevier	http://www.journals.elsevier.com	Child AND mountain OR highland
Nature Publishing Group	http://www.nature.com	Child AND mountain OR highland
Science Direct	http://www.sciencedirect.com	Child AND mountain OR highland
Chronic Poverty Research Centre, Manchester University	http://www.chronicpoverty.org/search	Child AND mountain OR highland
Childhood Poverty Research and Policy Centre (CHIP)	http://www.childhoodpoverty.org/index.php	Mountain OR highland
*UN Agencies*		
UNICEF	http://www.unicef.org/search	Mountain OR highland
World Health Organization	http://apps.who.int/iris/	Child AND mountain OR highland
ILO	http://www.ilo.org/global/publications/books	Child labour / labor
UNESCO	http://www.unesco.org/new/en/unesco/resources/publications/unesdoc-database/	Child AND mountain OR highland
UNDP	http://www.undp.org/content/undp/en/home/librarypage.html	Child AND mountain OR highland
World Bank	http://www.worldbank.org/reference/	Child AND mountain OR highland
Food and Agricultural Organization.	http://www.fao.org/publications/search/en/	Mountain
*NGOs*		
Human Rights Watch	http://www.hrw.org/publications/reports	Child AND mountain OR highland
Plan	http://plan-international.org/about-plan/resources/publications	Mountain OR highland
Oxfam GB	http://policy-practice.oxfam.org.uk/publications	Child AND mountain OR highland
Save the Children	http://www.savethechildren.org.uk/resources/online-library	Mountain OR highland
